# Leaf Extract from *Lithocarpus polystachyus* Rehd. Promote Glycogen Synthesis in T2DM Mice

**DOI:** 10.1371/journal.pone.0166557

**Published:** 2016-11-28

**Authors:** Jinfei Wang, Yumin Huang, Kaixiang Li, Yingying Chen, Diana Vanegas, Eric Scott McLamore, Yingbai Shen

**Affiliations:** 1 College of Biological Sciences and Technology, Beijing Forestry University, Beijing, China; 2 Guangxi Forestry Research Institute, Nanning, Guangxi, China; 3 Food Engineering Department, Universidad del Valle, Cali, Valle del Cauca, Colombia; 4 Agricultural and Biological Engineering Department, Institute of Agricultural and Food Science, University of Florida, Gainesville, Florida, United States of America; Institute of medical research and medicinal plant studies, CAMEROON

## Abstract

The purpose of this study was to investigate the effects of leaf extract from *Lithocarpus polystachyus* Rehd. on type II diabetes mellitus (T2DM) and the active ingredients of this effect. In addition, this study determined, for the first time, the underlying molecular and pharmacological mechanisms of the extracts on hyperglycemia using long-term double high diet-fed and streptozotocin (STZ) induced type II diabetic mice. In the present study, leaf extract, phloridzin and trilobatin were assessed *in vivo* (gavage) and *in vitro* (non-invasive micro-test technique, NMT) in experimental T2DM mice. The biochemical parameters were measured including blood glucose and blood lipid level, liver biochemical indexes, and hepatic glycogen. The relative expression of glycometabolism-related genes was detected. The effect of leaf extracts on physiological glucose flux in liver tissue from control and T2DM mice was also investigated. Body weight of experimental T2DM mice increased significantly after the first week, but stabilized over the subsequent three weeks; body weight of all other groups did not change during the four weeks’ study. After four weeks, all treatment groups decreased blood glucose, and treatment with leaf extract had numerous positive effects: a) promoted in glucose uptake in liver, b) increased synthesis of liver glycogen, c) reduced oxidative stress, d) up-regulation of glucokinase (GK), glucose transporter 2 (GLUT2), insulin receptor (IR) and insulin receptor substrate (IRS) expression in liver, e) down-regulation of glucose-6-phosphatase (G-6-P) expression, and f) ameliorated blood lipid levels. Both treatment with trilobatin or phloridzin accelerated liver glycogen synthesis, decreased oxidative stress and increased expression of GK. IRS and phosphoenolpyruvate carboxykinase (PEPCK) were both up-regulated after treatment with trilobatin. Expression of GLUT2, PEPCK and G-6-P were also increased in liver tissue after treatment with phloridzin. Our data indicate that leaf extract from *L*. *polystachyus* Rehd. has a preferable hypoglycemic effects than trilobatin or phloridzin alone. Leaf extract significantly increased glucose uptake and hepatic glycogen synthesis while also inducing a decline of hepatic gluconeogenesis and oxidative stress in T2DM mice. From this study, we draw conclusions that *L*. *polystachyus* promoted glycogen synthesis in T2DM mice, and that the active compounds were not only the trilobatin or phloridzin.

## 1. Introduction

Type II diabetes mellitus (T2DM) is now the third largest fatal disease, following only cancer and cardiovascular disease, and is one of the most challenging healthcare epidemics in the world [1–2]. The most common clinical manifestations of T2DM is high blood glucose levels. T2DM is a complex disorder characterized by abnormal metabolism of carbohydrates, lipids and proteins that results in the absolute or relative reduction of insulin activity [3]. Insulin resistance and pancreatic β cell dysfunction are causative factors and not the main mechanism of T2DM.

Insulin action is regulated by receptors on the surface of insulin-sensitive tissues, such as liver, adipose tissue and skeletal muscle [4]. Insulin signal transduction is initiated when insulin receptors (IR) are activated, providing docking sites for insulin receptor substrate (IRS) proteins that transduce insulin signaling to phosphatidylinositol (PI) 3-kinase [1]. In T2DM, plasma glucose clearance is relatively inefficient, inducing insulin resistance; within liver tissues this eventually results in altered metabolic gene expression and impaired glycometabolism [5], contributing to fasting hyperglycemia [6]. There is recent evidence that protein tyrosine phosphatase 1B (PTP1B) activity is the pathogenesis of insulin resistance [1].

Liver is a key regulator of glucose homeostasis [7], and converting glucose to glycogen is the primary fate of ingested glucose [8–9]. Plasma glucose levels are largely determined by the rate of glucose/glycogen turnover in the liver (primarily) and also in skeletal muscle [8]. Glycogen synthase is the rate-limiting enzyme for this process, which is encoded by two genes: GYS2, primarily expressed in liver, and GYS1, expressed in muscle and other tissues [8].

There are various western medicines to treat T2DM which can be divided into 5 main types as discussed, including drugs that: a) stimulating insulin production, b) reducing hepatic glucose production, c) delaying carbohydrate uptake in the gastrointestinal tract gut, d) improving insulin action, or and e) targeting the glycogen-like peptide receptor GLP-1 axis [10]. Although the current pharmacological interventions for the treatment of T2DM are effective, these medications are often associated with adverse side effects such as a slightly increasing risk of bladder cancer [11–13]. The fact is that the current medicines on the market are not 100% effective, which requested to find new active principles from medicinal plants [14].

Flavonoids are plant secondary metabolites that are now considered potential peripheral targets in the treatment of TD2M and other diseases. Flavonoids from medicinal plants are known to have hypoglycemic effects, which has been shown with soybean isoflavone [15], buckwheat leaf [16], mulberry leaf [17], *Momordica grosvenori* fruit [18], and *Lithocarpus polystachyus* leaves/tea [19]. Many flavonoids have been isolated from *L*. *polystachyus*, including phloretin/phlorizin, trilobatin, and quercetin [20].

Flavonoids, including phloridzin (an SGLT1/SGLT2 competitive inhibitor) and trilobatin (an inhibitor against α-glucosidase) have the ability to sustain normoglycemia in T2DM [21–23], but are not involved in hepatic glycogen synthesis. Thus, this study examined hepatic glycogen to validate whether leaf extract, phloridzin and trilobatin contribute to glucose homeostasis in T2DM by increasing hepatic glycogen synthesis. In addition, we also investigated the molecular and biochemical effects of leaf extract, phloridzin and trilobatin, on liver tissue to clarify the mechanisms of anti-hyperglycemic efficacy.

## 2. Materials and Methods

### 2.1 Materials

Kits for measuring total cholesterol (TC), triglyceride (TG), high-density lipoprotein cholesterol (HDL-C), low-density lipoprotein cholesterol (LDL-C), urea nitrogen (UN), malondialdehyde (MDA), total-superoxide dismutase (T-SOD), glutamate pyruvate transaminase (GPT), and glutathione (GSH) were purchased from Nanjing Jiancheng Chemical Factory (Nanjing, China). Collagenase I and IV, and streptozotocin (STZ) were purchased from Sigma (Beijing, China). TRlzol Reagent was purchased from Invitrogen (Carlsbad, CA). cDNA (RR036A) and quantitative real-time PCR kits (RR420A) were purchased from Takara (Shiga, Japan). Chloroplatinic acid, lead acetate, cerium (IV) oxide nanoparticle solution (<25 nm particle size, 10% with H_2_O), Nafion-117 solution, glucose oxidase (GOx), and glutaraldehyde were purchased from Sigma Aldrich (Atlanta, GA USA). Glibenclamide (a conventional medicine for T2DM and a type of Western medicine that stimulates insulin production, as a positive control in this study, which was used to distinguish Chinese tradition medicine and leaf extract) was purchased from Tianjin Pacific Pharmaceutical Co., Ltd. Portable blood glucose meter (SUPER GLUCOCARD II GT-1640, Jingdu, Japan), high speed refrigerated centrifuge (Sigma3-30K), UV spectrophotometer (4802 UV/VIS double beam spectrophotometer), microplate reader (Bio-Rad, US), CO_2_ incubator (CP-T), ultralow temperature freezer (MDF-382E(N)), pulverizer (DJ-10A, Shanghai), supersonic cleaner (USC-502, Bolong, Shanghai), rotary evaporators (laborota4000WB/G1, Shanghai), and macroporous resin (D801) were also used in this study. All chemicals were reagent grade from commercial source as noted.

### 2.2 Leaf extract from *Lithocarpus polystachyus* Rehd. plant

The leaf extract, phloridzin and trilobatin of *Lithocarpus polystachyus* Rehd. were obtained from Shanghai Institute of Materia Medica, Chinese Academy of Sciences. The leaves were collected in August 2012 from Bama Yao Autonomous County, Guangxi Zhuang Autonomous Region, People’s Republic of China, and identified by Mr. Zhonghua Liu of Beijing Forestry University. A voucher specimen has been deposited at the Herbaria of Beijing Forestry of University (BJFU-2012-GX-Fagaceae-13-1).

Dry leaves, 100g, of *L*. *polystachyus* Rehd. were powdered, and ultrasonic extracted twice with 1.0 L distilled water (25°C, 30min). The aqueous extract was distilled through reduced pressure distillation (60°C, 100MPa) and freeze-dried to get leaf extract [24]. The process was shown in S1 Fig. Simultaneously, the same of the aqueous extract were filtered with 0–90% microporous resin, and collected 40–60% section. This section was re-melted with 30–50% methanol-water (50–100 mL), and standed for one night, then recrystallized respectively to get 5g phloridzin and trilobatin respectively [25]. Of course, the purity and structure of phloridzin and trilobatin were detected with 400M Hz ^1^H NMR (Varian, US). The results were shown in S2 Fig.

### 2.3 Animals and groups

Clean grade male Kunming mice, 20±2 g, were purchased from Academy of Military Medical Science. The mice were kept in a separate room as the following conditions: 22±2°C, 50±10% relative humidity, 12/12 h light-dark cycle [26], with free access to water. The 60 mice were acclimatized for 1 week, and randomly divided into two groups: 1) standard feed group, n = 10 mice, and 2) high sugar/high fat group [27–28], n = 50 mice. The ingredients for the high sugar & high fat mice feed are shown in S1 Table. The studies were approved by the Animal Ethics Committee of Beijing Forestry University.

After four weeks, the high sugar/high fat group were fasted 12 h (from 20:00PM to 8:00AM at next day), and then were given a single injection of STZ (60mg/kg body weight, 0.1mL/10g body weight) [27] dissolved in pre-chilled citrate buffer (pH = 4.2) [29]. On the 7^th^ day after injection, the tail vein fasting blood glucose was determined using a portable blood glucose meter. Mice with blood glucose levels of at least 11.1mmol/L were considered experimental type II diabetes (T2DM) mice. These experimental T2DM mice were randomly divided into 5 subgroups: i) T2DM group, and mice treated with ii) glibenclamide (80mg/kg), iii) leaf extract (0.8 g/kg), iv) phloridzin (80 mg/kg), or v) trilobatin (80 mg/kg) for four weeks. The standard feed group (referred to as control group) and T2DM group were gavage tap water for successive 4 weeks. The gavage doses were 0.1 mL/10g/d for all the mice once daily at 9:00AM. Thus, there are six groups including control, T2DM, glibenclamide, leaf extract, phloridzin, and trilobatin groups in this study.

### 2.4 Body weight and blood glucose

Body weights were measured on an electronic scale (maximum range: 200 g, precision: 0.01 g) once a week at the 7^th^ day (after injection, before gavage), 14^th^ day (gavage one week), 21^th^ day (gavage two weeks), 28^th^ day (gavage three weeks), and 35^th^ day (gavage four weeks) [30]. Simultaneously, blood glucose was measured by tail vein after fasted 2 h.

### 2.5 Oral glucose tolerance test (OGTT) and area under the curve of blood glucose (AUCG)

At the end of four weeks’ gavage, all mice were fasted for 12 h (from 20:00PM to 8:00AM at next day), and 2 g/kg (body weight) glucose solution [28] was administered orally using gavage [31]. Samples of blood glucose were collected from the tail vein at 30, 60 and 120 min after gavage. The area under the curve of blood glucose (AUCG) was calculated as described by Gan *et al*. [32].

### 2.6 Blood biochemical measurement (lipids)

On the 35^th^ day, mice were fasted for 12 h, and then anesthetized by ethyl ether. Blood samples were collected into plastic vacuum blood collection tubes by ocular enucleation, centrifuged (3500 rpm, 10 min, 4°C) to get serum, and then stored at -20°C. TC, TG, HDL-C, LDL-C and UN were measured using commercial kits and a UV spectrophotometer. GPT was measured using commercial kits and the microplate reader.

### 2.7 Liver biochemical indexes measurement

After collecting blood samples, mice were killed immediately by cervical dislocation and liver tissue was removed promptly and weighted. A portion of tissue was grinded within liquid nitrogen, homogenized with cold saline, and centrifuged (2500 rpm, 10 min, 4°C) to get supernatant for future measurements. Glycogen, MDA, T-SOD and GSH were measured using a UV spectrophotometer according to instructions of the commercial kits. Another portion was stored for molecular experiments.

### 2.8 Liver small tissue culture

Liver tissue was cut into pieces, and enzymolysis was carried out using collagenase I and collagenase IV (Quality ratio is 1) 5 mL at 37°C for 45 min in a digital circulating water bath with intervals of 15min shaking. The supernatant was removed and 10mL of precooled Hank’s buffer (8 mg KCl, 6 mg CaCl_2_, 0.35 g NaHCO_3_, 6 mg KH_2_PO_4_, 0.06 g Na_2_HPO_4_ ·12 H_2_O, 8 g NaCl, 1 g glucose in 1L DI water) at 4°C was added to terminate enzymolysis [33, 34]. Then, similar-sized small liver tissue was selected in the black background using an anatomical lens, and tissue preps were incubated in RPMI 1640 culture media with 5% CO_2_.

### 2.9 Glucose biosensor flux measurements

Glucose micro biosensors were fabricated based on McLamore *et al*. [35] and Chaturvedi *et al*. [36]. Briefly, nanoplatinum was electrodeposited on platinum/iridium microelectrodes (PI20033.0A10, 51mm length, 0.256mm shaft diameter, 1–2 μm tip diameter, 3 μm parylene C coated metal shaft) in solution of 0.728% chloroplatinic acid and 0.002% lead acetate at 10 V for 30 s. Next, the tip of the microelectrode was immersed in cerium (IV) oxide nanoparticle solution for 5 min, and then air dried at room temperature for one hour. The electrode was then dipped in Nafion solution for 20 min to form an anion-exclusion membrane and dried in an oven at 115°C for 1 hour. To impart specificity for glucose, the nanomaterial-modified electrode was dipped in a solution of 50mg GOx/mL PBS for 30 min at 4°C. Finally, the biosensor was immersed in glutaraldehyde for 10s to cross link the protein, and air dried for 10min in air at room temperature. For all biosensor experiments a working potential of +500 mV was used. All glucose biosensors were calibrated in Hanks’ buffer prior to use based on McLamore *et al*. [35]. The working range was defined as the linear sensing range where the R^2^ value for linear calibration plots was > 0.98.

Net flux of glucose [37] was measured using the non-invasive micro-test technique (NMT; also called the self-referencing microelectrode technique; see McLamore for details on the technique) [35]. Small liver tissue samples were soaked in Hanks’ buffer in a 35 mm Petri dish at pH 7.4 for 30 min [38–39]. Then, a glucose microsensor was positioned at the tissue surface (within 1–2 μm), using an inverted microscope and computer controlled 3D linear actuators. The differential current (Δi) was recorded during oscillation of the microsensor with an excursion distance (*ΔX*) of 30 μm. After conversion of the oxidative current to glucose concentration (via the calibration curve), glucose flux (*J*) was calculated using Fick’s first law of diffusion [35, 37, 40]:

$$J=D\frac{\Delta C}{\Delta X}=D\frac{C_{1}-C_{2}}{\Delta X}$$ (1)

where: *J* is the glucose flux (pmol cm^-2^ s^-1^), *D* is molecular diffusion coefficient for glucose (1.50×10^−6^ cm^2^ s^-1^), Δ*C* is the measured glucose concentration gradient (pmol ml^-1^), and Δ*X* is the excursion distance for the microelectrode oscillation (μm). After recording a baseline flux, a 100uL aliquot of 50 mM glucose was added to the dish and glucose flux was measured. After 20 min, an aliquot of 100 uL of 32 mg/mL drug (as noted) was added while measuring glucose flux. Each tissue prep was measured once, and the experiment was repeated at least three times.

To analyze the effect of glucose stimulation and extract addition on net flux, glucose flux data was collected in real time for at least 5 min, and then 20 mM exogenous glucose was added to Hanks’ buffer; finally, extract was added. For each step in the process, the steady state glucose flux was used to assess the effect(s) net glucose transport. The steady state parameters were: i) mean baseline flux (*J*_*basal*_), (ii) average glucose flux after stimulating with 20 mM exogenous glucose (*J*_*glucose*_), and (iii) the glucose flux after addition of 20 mM glucose and drug (*J*_*post*_). The effect of glucose stimulation on net flux (*ΔJ*_*stim*_) was calculated as follows:

$$\Delta J_{stim}=\frac{J_{glu\text{cos}e}-J_{basal}}{J_{basal}}$$ (2)

The effect of extract addition relative to baseline flux (*ΔJ*_*medicine*_) was calculated as follows:

$$\Delta J_{medicine}=\frac{J_{post}-J_{basal}}{J_{basal}}$$ (3)

The effect of extract addition relative to stimulated flux (*ΔJ*_*medicine_stim*_) was calculated as follows:

$$\Delta J_{medicien\_stim}=\frac{J_{post}-J_{glu\text{cos}e}}{J_{glu\text{cos}e}}$$ (4)

### 2.10 Quantitative real-time polymerase chain reaction (qRT-PCR)

The total RNA of six groups was extracted from less than 100mg of liver tissue using TRlzol reagent. RNA quality and concentration were assessed using a WPA spectrophotometer (OSTA, China) and 1.5% agarose gel electrophoresis. According to the concentration, reverse transcription was performed on 3 mg of total RNA using a high-capacity cDNA kit. Primers used in qRT-PCR are shown in S2 Table. Quantitative real-time PCR was performed in a Bio-Rad CFX96^™^ Real-Time System (C1000 Touch Thermal Cycler). The mRNA levels of all genes were normalized using GADPH as internal control [41–42].

### 2.11 Statistical analysis

Normal or repeated measures ANOVA analyses were carried out using SPSS 12.0 software, followed by LSD or Dunnett's multiple comparisons tests. Values were considered significantly different when the *P* value was less than 0.05. All results are represented as mean ± SEM.

## 3. Results

### 3.1 Body weight and blood glucose

**Fig 1:**
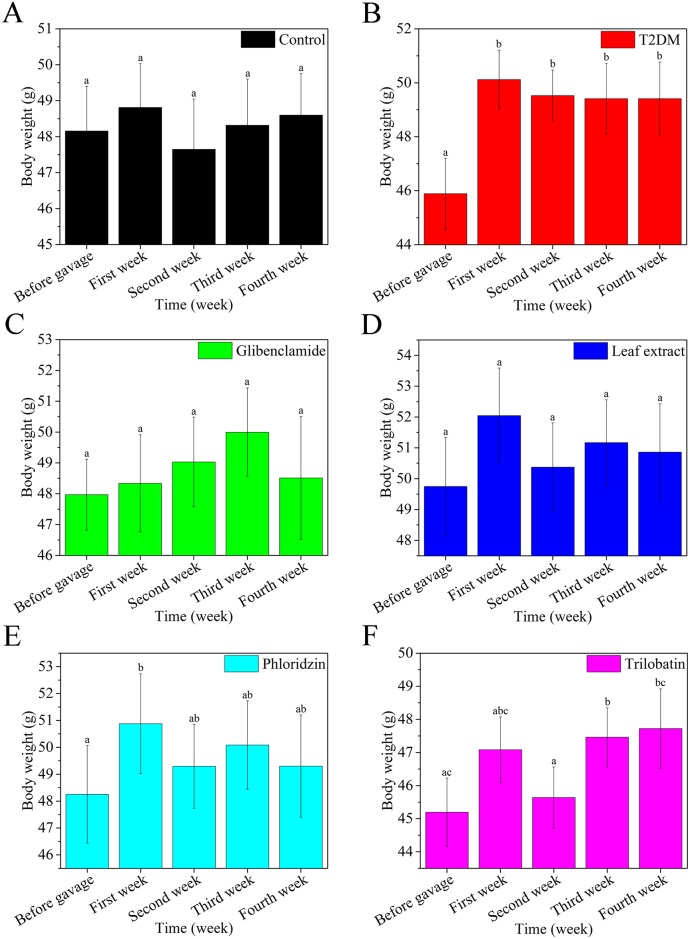
Leaf extracts from *L*. *polystachyus* Rehd. have no effect on body weight of mice after four weeks of gavage. Body weight of groups control (A), glibenclamide (C), leaf extract (D) and trilobatin (F) were relatively unchanged, while body weight of the T2DM group (B) increased significantly after the first week and stabilized over the subsequent three weeks. As well as phloridzin (E) slightly increased at first week and fluctuated after the continue weeks. The data were analyzed using an ANOVA with repeated measures with a Sphericity Assumed or Greenhouse-Geisser correction, and the mean scores for body weight were statistically reported that control (*F* (4, 36) = 2.312, *P* = 0.076), T2DM (*F* (1.467, 14.675) = 6.556, *P* = 0.014), glibenclamide (*F* (2.169, 21.686) = 0.802, *P* = 0.471), leaf extract (*F* (1.505, 15.054) = 2.721, *P* = 0.109), phloridzin (*F* (4, 40) = 5.01, *P* = 0.002) and trilobatin (*F* (2.606, 26.058) = 6.228, *P* = 0.003), respectively. All values are expressed as the mean ± SEM, n = 10. Columns labeled with different letters are significantly different at *P* < 0.05.

To test the effects of *L*. *polystachyus* Rehd. leaf extract on body weight, all the groups were tested before and after treatment. Body weight was monitored weekly (Fig 1). Compared with before gavage, the body weight of all groups did not change significantly, with exception of TD2M mice—which increased after the first week and then stabilized over the subsequent three weeks, and trilobatin—which slightly increased at the first week and fluctuated after the continue weeks.

**Fig 2:**
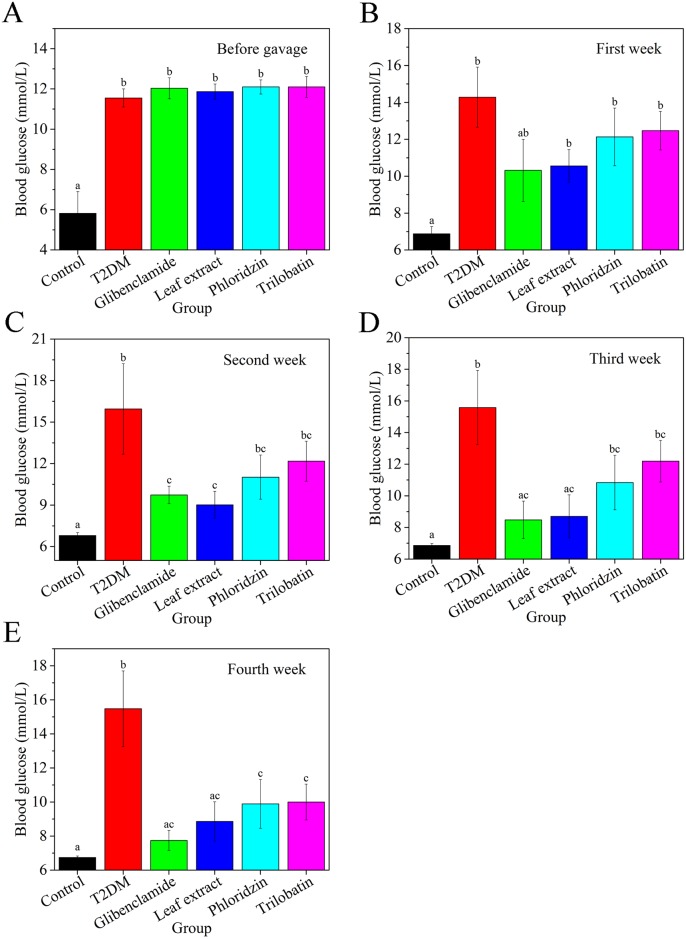
Leaf extracts from *L*. *polystachyus* Rehd. decrease blood glucose from tail of experimental T2DM mice after gavage at different weeks. (A) was Before gavage, (B) was the First week after treatment, (C) was the Second after treatment, (D) was the Third week after treatment and (E) was the Fourth week after treatment. The data were analyzed with ANOVA. All the values are expressed as the mean ± SEM, n = 10. Columns labeled with different letters are significantly different at *P* < 0.05.

Fig 2 shows the hypoglycemic effect on experimental T2DM mice for various diet groups over a four-week period. Before gavage, T2DM mice and all treatment groups had significantly higher blood glucose than control mice. There was no significant different among the treatment and T2DM groups at before gavage (Fig 2). After one-week gavage, T2DM mice, and groups of leaf extract, phloridzin and trilobatin had significantly higher blood glucose than the control group, but mice treated with glibenclamide did not. After two weeks, all the groups had higher blood glucose levels compared to the control group; there was no difference among the groups that had high blood glucose. Mice treated with glibenclamide and leaf extract had markedly lower levels compared with T2DM mice, while both phloridzin and trilobatin showed little difference compared to T2DM mice. After three weeks, blood glucose was not different than the control group for mice treated with glibenclamide or leaf extract, and significantly lower than T2DM mice; the phloridzin and trilobatin groups were not significantly different than the T2DM mice. At four weeks post gavage, all groups were similar to control group, particularly the glibenclamide and leaf extract groups. Blood glucose of groups control, T2DM, phloridzin and trilobatin were relatively stable over the four weeks’ treatment. Blood glucose of glibenclamide and leaf extract groups gradually decreased, and after three weeks, the levels were similar to normal levels observed for control mice. Over the four weeks’ treatment, blood glucose of mice treated with phloridzin slowly decreased, but the levels at fourth week were still higher than control mice. However, there was no significant downward trend in trilobatin group over four weeks’ treatment (S3 Fig).

### 3.2 OGTT and AUCG

**Fig 3:**
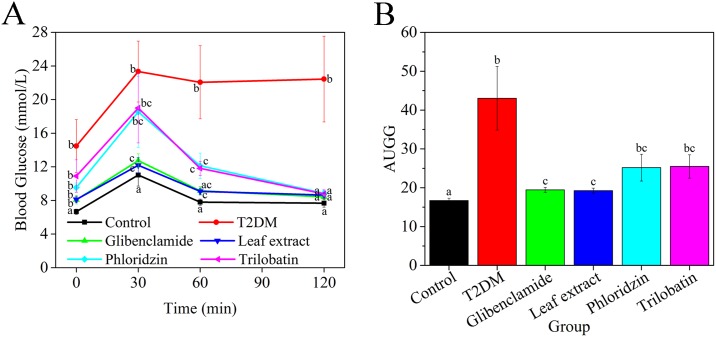
Effect of leaf extracts from *L*. *polystachyus* Rehd. on glucose tolerance. (A) OGTT tests for treatment groups after four weeks’ gavage; data taken after 12 h fast from tail at 0, 30, 60 and 120 min after glucose loading. (B) AUCG of mean blood glucose for all treatments. The data were analyzed with ANOVA. All values are expressed as the mean ± SEM, n = 10. Columns labeled with different letters are significantly different at *P* < 0.05.

The oral glucose tolerance test (OGTT) was used as a screening method for acute anti-hyperglycemic activity, which is a standard method to examine regulation of blood glucose [31]. The area under the curve of blood glucose (AUCG) test was used to evaluate net drug utilization [43]. Our results (Fig 3) indicate that experimental T2DM mice treated with leaf extracts of *L*. *polystachyus* Rehd. and glibenclamide had improved regulation of blood glucose. Phloridzin and trilobatin improved glucose intolerance induced by the high-fat diet based on OGTT, but to a lesser degree than the leaf extract. Blood glucose of all groups increased to a peak value at 30 min after glucose loading, and then decreased to stable levels. The glibenclamide (12.74 ± 0.81 mmol/L) and leaf extract (12.18 ± 0.91 mmol/L) groups were closed to control mice (11.02 ± 1.30 mmol/L) at 30 min after glucose loading, but phloridzin (18.54 ± 0.43 mmol/L) and trilobatin (19.00 ± 4.15 mmol/L) groups were significantly higher, and similar to the T2DM group (23.34 ± 3.63 mmol/L). After one hour of glucose loading, blood glucose of all groups declined relative to the peak value. After one hour the T2DM group (22.06 ± 4.35 mmol/L) was significantly higher than control mice (7.80 ± 0.37 mmol/L), glibenclamide (9.14 ± 0.52 mmol/L) and leaf extract (9.08 ± 0.38 mmol/L) groups. After two hours post loading, blood glucose of T2DM mice (22.44 ± 5.10 mmol/L) was not remarkably different than the peak value, while all other groups were similar to control mice (7.68 ± 0.48 mmol/L). The AUCG data (Fig 3B) indicates two major responses from the treatment groups: mice treated with glibenclamide (19.46 ± 0.64) or leaf extract (19.25 ± 0.65) were not significantly different than control mice (16.7 ± 0.62); while average AUCG for phloridzin (25.18 ± 3.44) or trilobatin (25.48 ± 3.02) treated mice was significantly higher than control mice, but lower than T2DM mice (43.06 ± 8.21).

### 3.3 Liver glycogen

**Fig 4:**
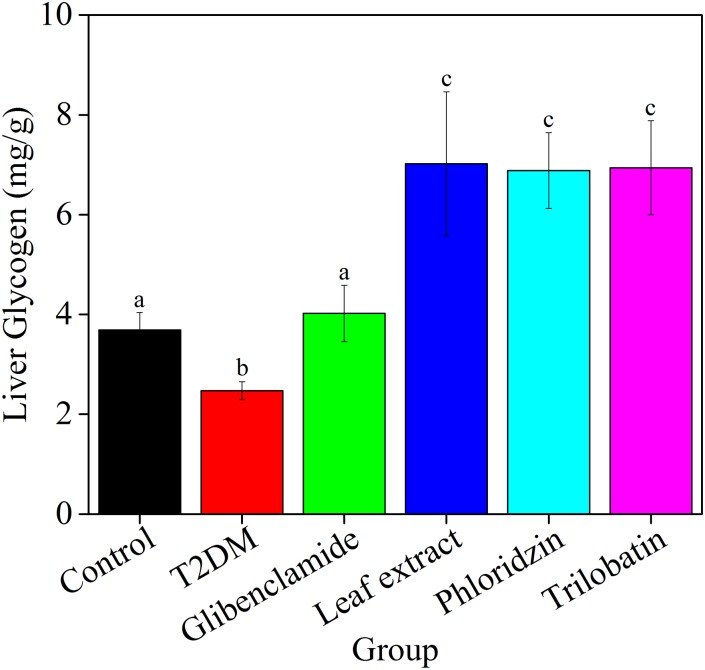
Leaf extracts from *L*. *polystachyus* Rehd. significantly increase liver glycogen of experimental T2DM mice four weeks post gavage. After 12 h fast, liver samples were immediately separated, dipped into liquid nitrogen and stored in -80°C prior to measure liver glycogen. The data were analyzed with ANOVA. All the values are expressed as the mean ± SEM, n = 10. Columns labeled with different letters are significantly different at *P* < 0.05.

To further understand hypoglycemic effects induced by increased liver glycogen, the content of hepatic glycogen was measured for all groups four weeks post gavage (Fig 4). Liver glycogen for experimental T2DM mice (2.47 ± 0.18 mg/g) was significantly lower (*P* < 0.05) than control mice (3.69 ± 0.34 mg/g). With the exception of glibenclamide treatment (4.02 ± 0.56 mg/g), liver glycogen levels of all groups increased significantly (leaf extract = 7.02 ± 1.44, phloridizin = 6.88 ± 0.76 and trilobatin = 6.94 ± 0.94 mg/g) over the four-week treatment.

### 3.4 Glucose flux

**Fig 5:**
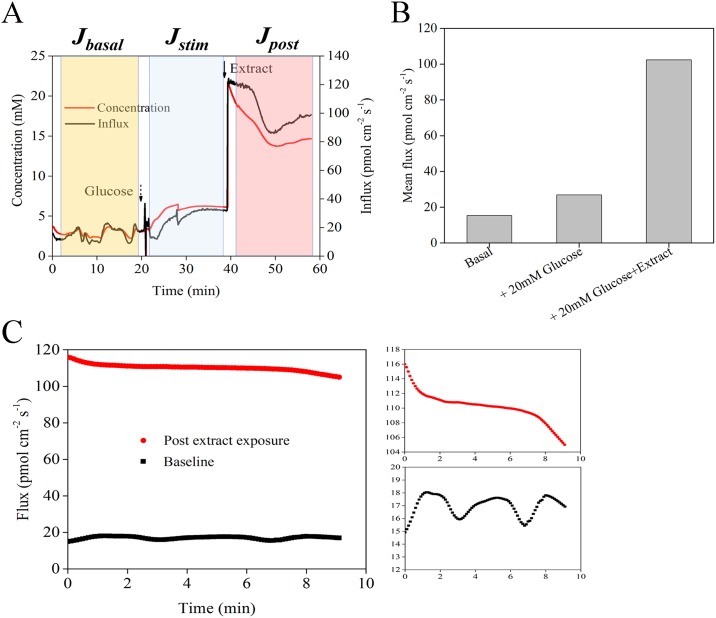
A representative example of glucose flux and concentration, and mean flux. (A) Representative sample showing glucose influx for a tissue taken from the flavonoid group (no exogenous insulin added). After addition of 20 mM exogenous glucose (shown by a dashed arrow), glucose uptake gradually increased (blue highlighted section). After addition of drug, an immediate and significant increase in glucose flux occurred, and flux stabilized at this level (red highlighted section). (B) Mean flux of glucose in *J*_*basal*_, *J*_*stim*_, and *J*_*post*_. (C) Representative glucose oscillations before and after addition of glucose+extract. Prior to exogenous glucose addition, regular ultradian oscillations in glucose flux, but these waves were dampened after addition of glucose/extract.

**Fig 6:**
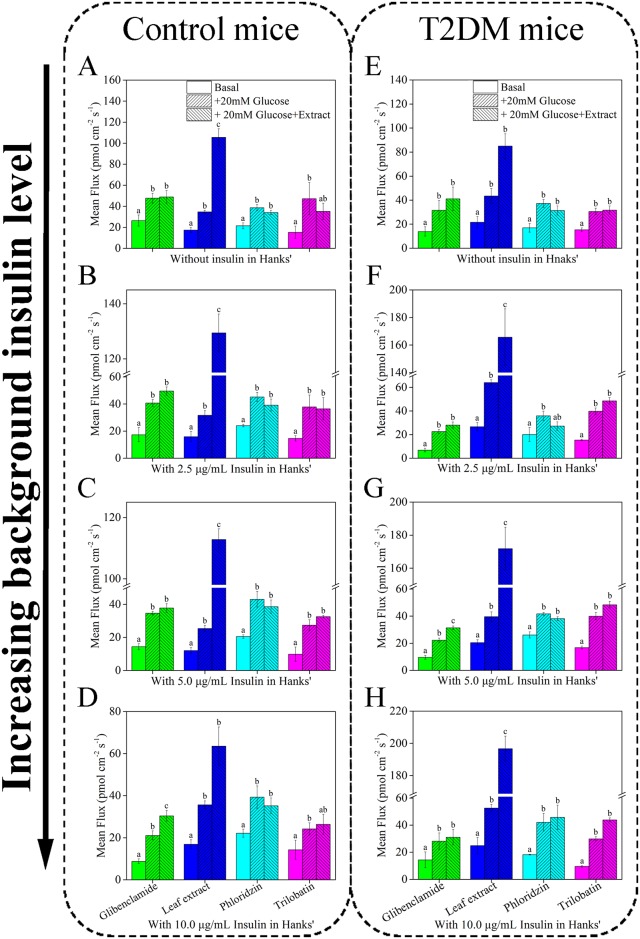
Effect of leaf extracts from *L*. *polystachyus* Rehd. on mean glucose influx of small liver tissue. A-D shows glucose influx of control mice with different level insulin in Hanks’, E-H shows glucose influx of experimental T2DM mice. Different colors represent four treatment groups. The data were analyzed with ANOVA. The values are expressed as the mean ± SEM, n = 6. Columns labeled with different letters are significantly different at *P* < 0.05.

To further explore the underlying mechanism related to the observation of decreased glucose with increased glycogen synthesis levels in Figs 2–4, glucose uptake (i.e., influx) at the tissue surface was measured using the NMT technique. Fig 5A shows a representative plot from one of the measurements taken from the leaf extract group in the absence of insulin. The baseline glucose flux followed regular ultradian oscillations (the inset in Fig 5C shows an exploded view of the oscillations). The mean baseline glucose flux for all experimental T2DM mice was 14.9 ± 1.3 pmol cm^-2^ sec^-1^, the amplitude of oscillations was 1.5 ± 0.4 pmol cm^-2^ sec^-1^, and the period of the rhythmic wave was 2–3 min; all measurements are similar to oscillations observed by Shi (b) *et al*. [44]. For control mice, the average oscillation period (4–6 min) was significantly longer (see S4 Fig for details). After addition of 20 mM excess glucose (shown by a dashed in Fig 5A), the glucose waves were dampened, and glucose flux gradually increased, which demonstrates that tissues were metabolically active and responsive to glucose stimulation; this dampening is similar to Shi (c) *et al*. [45] and McLamore *et al*. [35]. After at least 30 secs of no significant change in flux, extract was added to the dish (noted by an arrow in Fig 5A); this caused an immediate and significant increase in glucose flux. In preliminary experiments, oscillations resumed after approximately 20 min when the concentration decreased to near basal levels (data not shown). In order to minimize cell stress during these experiments, extract was added prior to the onset of oscillations. After approximately five minutes of stable readings (post glucose stimulation), extract was added to the dish, which caused a pronounced increase in glucose influx for the sample shown in Fig 5. Approximately five min after the peak in glucose influx, oscillatory behavior continued and persisted for at least 30 min (oscillation data not shown for brevity). Like shown in Fig 5B, in order to statistical analysis, we calculated mean flux in *J*_*basal*_, *J*_*stim*_, and *J*_*post*_ (the later flux data were calculated by this way) [44]. Because in Fig 5B, just one sample was measured, there was no error bar. The mean glucose flux under basal, exogenous glucose, and glucose plus extract is discussed in detail below (Fig 6).

Normally, blood glucose is elevated after a meal, which results in a concomitant increase in blood insulin levels. For T2DM, there is a higher concentration of blood insulin than normal due to insulin resistance. Based on this, the experiment in Fig 5 was repeated in the presence of various insulin levels: 0 μg/mL, 2.5 μg/mL (representing lower than physiological conditions), 5.0 μg/mL (normal levels), and 10.0 μg/mL (elevated levels). First, glucose flux for tissues from control mice (Fig 6A–6D), and experimental T2DM mice (Fig 6E–6H) were measured in Hanks’ buffer.

The data from control mice in Fig 6A–6D shows that for all levels of insulin tested: 1) addition of exogenous glucose (20mM) induces glucose influx, 2) glibenclamide (a medicine that stimulates insulin production) has little effect on glucose uptake except 10μg/mL insulin level, 3) leaf extract caused a dramatic increase in glucose influx, as much as four times higher flux levels than addition of exogenous glucose under 5.0 μg/mL background insulin, 4) phloridzin, an SGLT1/SGLT2 competitive inhibitor, blocked glucose uptake in control mice at all concentrations of insulin, and 5) trilobatin, an inhibitor against α-glucosidase, did not cause a change in glucose flux for any background concentration of insulin. For T2DM mice (Fig 6E–6H) some marked differences were noted compared to control mice, namely: 1) glibenclamide caused an increase in glucose influx with 5.0 μg/mL background insulin, which aligns with the sulphonylurea theory—together with insulin, sulphonylureas improve insulin section and decrease blood glucose, 2) leaf extract caused a dramatic increase in glucose uptake, but the most pronounced effect (as much as four times higher influx than addition of exogenous glucose) occurred with background insulin levels of 5.0 μg/mL, 3) phloridzin inhibited glucose uptake, and 4) trilobatin enhanced glucose influx but only at background insulin levels of 10.0 μg/mL.

Comparing flux for the different background insulin levels, addition of exogenous glucose (20mM) significantly increased glucose uptake for both control and T2DM mice at all insulin levels. When there was no background insulin, leaf extract increased glucose uptake. When insulin levels were low (2.5 μg/mL) treatment with leaf extract increased glucose influx by 3–4 times over other treatments in both control and T2DM mice. With normal insulin levels (5.0 μg/mL), glibenclamide and leaf extract treatment enhanced glucose uptake in T2DM mice, although the effect was significantly greater for the leaf extract group; in control mice, only leaf extract significantly increased glucose uptake. When insulin levels were elevated (10.0 μg/mL), leaf extract again had the most pronounced effect on glucose uptake, while trilobatin caused a relatively small increase in glucose uptake for T2DM mice, but not for control mice.

**Fig 7:**
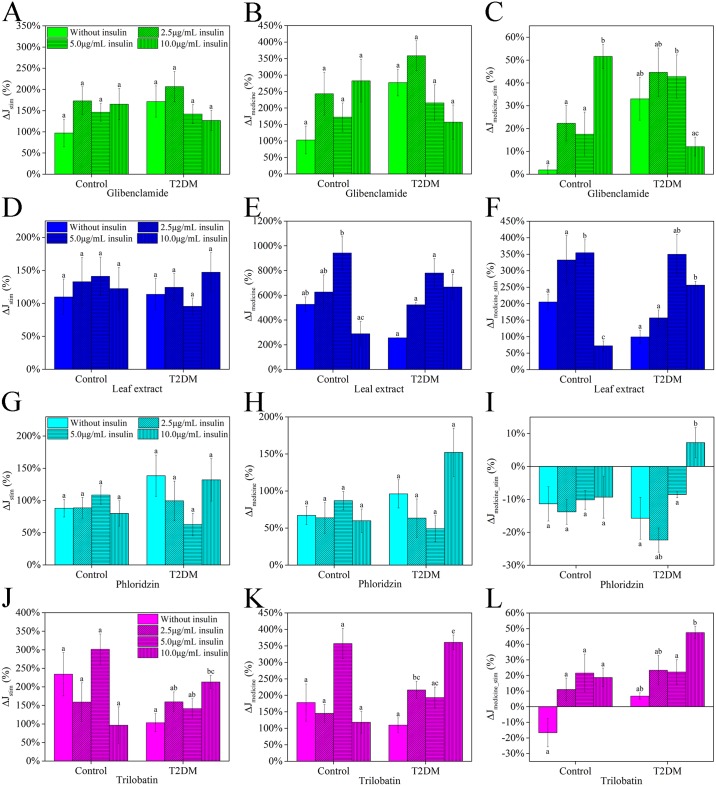
Effect of leaf extracts from *L*. *polystachyus* Rehd. on ΔJ of glucose mean influx. Different colors mean different treatment, and different shapes mean different level of insulin in Hanks’. First column shown *ΔJ*_*stim*_, second column shown *ΔJ*_*medicien*_, and third column shown *ΔJ*_*medicine_stim*_. The data were analyzed with ANOVA. The values are expressed as the mean ± SEM, n = 6. Columns labeled with different letters are significantly different at *P* < 0.05.

Naturally, there were individual differences among mice, and additional data analysis was required to gain a deeper understanding of the effect of individual extracts on glucose flux. To analyze the effect of glucose stimulation on flux, data was collected following the protocol in Fig 7, and the following three parameters were calculated from the data: i) the effect of glucose stimulation on net flux (*ΔJ*_*stim*_) was calculated as follows, ii) the effect of medicine addition relative to baseline flux (*ΔJ*_*medicine*_) was calculated as follows, and iii) the effect of extract addition relative to stimulated flux (*ΔJ*_*medicine_stim*_) was calculated as follows. Fig 7A, 7D, 7G and 7J show the data for, *ΔJ*_*stim*_, which indicate that stimulation by glucose promotes glucose uptake in both control and experimental T2DM mice. For control mice, the highest *ΔJ*_*stim*_ value was with normal insulin background levels (5.0 μg/mL), except glibenclamide. The value of *ΔJ*_*stim*_ was lower with 2.5 μg/mL background insulin, but when there was no background insulin or excessive levels (10.0 μg/mL), the *ΔJ*_*stim*_ reduced significantly such as leaf extract group. Fig 7B, 7E, 7H and 7K show the values of (*ΔJ*_*medicine*_) for different treatment groups. With the exception of leaf extract treatment, medicines had no effect on glucose uptake in control mice. Treatment with leaf extract increased (*ΔJ*_*medicine*_) with increasing insulin levels and was also maximum in the presence of normal background insulin (5.0 μg/mL). However, in the presence of 10.0 μg/mL insulin, the (*ΔJ*_*medicine*_) was significantly reduced. Analogously, in T2DM mice, added leaf extract had similar results with gradient insulin level. Nevertheless, with higher insulin level in Hanks’, rate also increased in T2DM mice and trend was lower than normal level. Fig 7C, 7F, 7I and 7L show *ΔJ*_*medicine_stim*_ data for various treatments. Glibenclamide had no impact on (*ΔJ*_*medicine_stim*_) for both control (except 10.0 μg/mL insulin) and experimental T2DM mice. Leaf extract treatment significantly increased *ΔJ*_*medicine_stim*_, for insulin levels from 0 to 5.0 μg/mL, but was suppressed when background insulin levels were high in control mice. Phloridzin significantly decreased (*ΔJ*_*medicine_stim*_) due to inhibition of SGLT2, but not in the presence of 10.0 μg/mL insulin. For trilobatin, there was no visible effects on (*ΔJ*_*medicine_stim*_).

### 3.5 qRT-PCR

Glucose transport into cells is the first step in glycogen synthesis in the liver; followed by transformation to glucose-6-phosphate (G-6-K) by glucokinase (GK), an isozyme of hexokinase. Meanwhile, insulin is recognized by IR, which upon activation provides docking sites for IRS proteins. mRNA relative expression is known to decrease after exogenous addition of glucose for T2DM [42, 46]. Under this condition, genes related to glycogen synthesis and genes inhibiting hepatic gluconeogenesis have also been reported [5]; for example, overexpression of phosphoenolpyruvate carboxykinase (PEPCK) and G-6-P.

**Fig 8:**
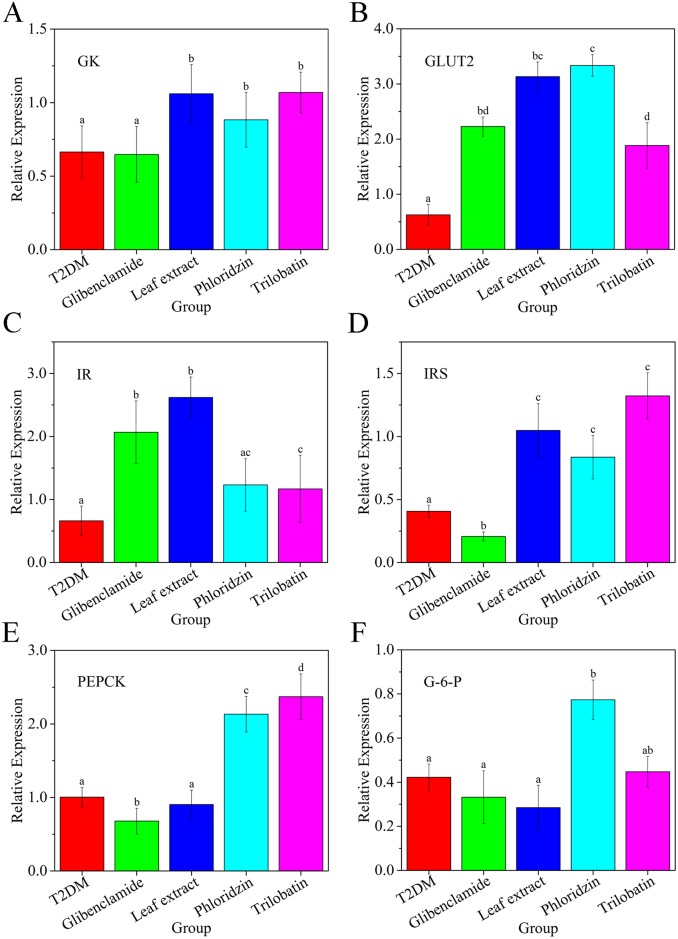
Effect of *L*. *polystachyus* Rehd. leaf extracts on relative expression for T2DM group. (A) glucokinase (GK), (B) glucose transport 2 (GLUT2), (C) insulin receptor (IR), (D) insulin receptor substrate (IRS), (E) phosphoenolpyruvate carboxykinase (PEPCK), and (F) glucose-6-phosphatase (G-6-P). The data were analyzed with ANOVA. Relative expression values are expressed as the mean ± SEM, n = 6. Columns labeled with different letters are significantly different at *P* < 0.05.

To validate these previous studies and evaluate the effect of *L*. *polystachyus* Rehd. leaf extracts, mRNA relative expression of GK, GLUT2, IR, IRS, PEPCK and G-6-P were measured in the liver using qRT-PCR analysis. Fig 8 shows that other than the glibenclamide group, relative expression of GK was increased in all groups compared with T2DM mice, with the most pronounced effect observed for the leaf extract group (Fig 8A). Relative expression of GLUT2 was significantly increased for all groups. Expression of IR and IRS increased for all groups with the exception of glibenclamide, which caused a decrease in IRS expression. PEPCK expression (Fig 8E) was unchanged after leaf extract, decreased after glibenclamide treatment, and increased after phloridzin or trilobatin treatment. The mRNA expression of G-6-P (Fig 8F) decreased after leaf extract treatment, increased for the phloridzin group, and did not change for the other treatment groups.

### 3.6 Liver biochemical indexes levels (MDA, T-SOD, GTP and GSH)

Hyperglycemia causes oxidative stress in T2DM [47], which in turn causes many chronic complications [48]. Polyphenols [49] and flavonoids [50] have been shown to alleviate oxidative stress in by scavenging oxygen free radicals and small molecules associated with antioxidant injury. T-SOD, GSH were usually regarded as an indicator of the health of the antioxidant [51]. MDA was an indicator of the lipid peroxidation process [51]. To examine the role of leaf extract in alleviation of oxidative stress in T2DM mice, we continually evaluated the impact of *L*. *polystachyus* Rehd. leaf extracts on liver oxidative stress (Table 1). Importantly, liver oxidative stress in the T2DM group were significantly increased compared with the control group. Following treatment with leaf extract and glibenclamide, all levels decreased significantly (this more pronounced after treatment with extracts than glibenclamide). Treatment with leaf extract, malondialdehyde and glutathione levels were similar to control mice. After four weeks of phloridzin treatment, glycosylated serum protein and superoxide dismutase levels decreased significantly; however, malondialdehyde levels did not change and glutathione levels increased. Following trilobatin treatment, glutathione levels were lower than control, and glycosylated serum protein levels slightly decreased; however, malondialdehyde and superoxide dismutase levels increased following.

**Table 1:** Hepatic biochemical indexes levels of malondialdehyde (MDA), superoxide dismutase (T-SOD), glutamate pyruvate transaminase (GPT), or glutathione (GSH) in mice following 4 weeks of treatment with glibenclamide, leaf extract, phloridzin, or trilobatin.

	Control	T2DM	Glibenclamide	Leaf extract	Phloridzin	Trilobatin
**MDA (nmol/mgprot)**	4.36±0.47a	6.50±0.88b	5.98±0.38bc	4.53±0.69abc	6.36±0.80bc	16.70±1.96c
**T-SOD (U/mgprot)**	81.31±5.13a	226.28±1.95b	177.27±5.04c	140.93±0.98c	173.97±1.65c	242.08±1.88c
**GPT (U/mgprot)**	174.07±29.43a	515.93±52.72b	389.03±23.07bc	260.72±38.74ac	240.47±24.01c	439.64±27.43bc
**GSH (mgGSH/gprot)**	0.0415±0.01ac	0.0648±0.01a	0.0453±0.00ac	0.0474±0.01ac	0.0733±0.01ac	0.0384±0.00bc

After four weeks’ treatment, hepatic biochemical indexes levels were performed. The data were analyzed with ANOVA. All the values are expressed as the mean ± SEM, n = 10. Columns labeled with different letters are significantly different at *P* < 0.05.

### 3.7 Blood biochemical levels (lipids)

Finally, we assessed the impact of *L*. *polystachyus* Rehd. leaf extracts on lipid levels (Table 2). T2DM mice had higher levels of all lipids compared to the control, with the exception of high-density lipoprotein cholesterol. After 4 weeks of treatment with leaf extract, levels of low density lipoprotein cholesterol and urea nitrogen were similar to control mice, while total cholesterol and triglyceride remained at elevated levels; the total cholesterol levels were high because the high density lipoprotein cholesterol increased significantly beyond baseline levels, even though low density lipoprotein levels decreased. Treatment with glibenclamide had a moderate positive effect, but lipid levels did not return to basal concentration. Phloridzin treatment caused no change in high-density lipoprotein cholesterol or triglyceride, but caused a decrease in low-density lipoprotein cholesterol and urea nitrogen. Trilobatin treatment caused an increase in triglyceride, high-density lipoprotein cholesterol, and urea nitrogen, but a decrease in low-density lipoprotein cholesterol.

**Table 2:** Lipid levels, including total cholesterol (TC), triglyceride (TG), high-density lipoprotein cholesterol (HDL-C), low-density lipoprotein cholesterol (LDL-C), and urea nitrogen (UN) in mice following 4 weeks of treatment with glibenclamide, leaf extract, phloridzin, or trilobatin.

	Control	T2DM	Glibenclamide	Leaf extract	Phloridzin	Trilobatin
**HDL-C (mmol/L)**	2.90±0.12a	2.16±0.19b	2.22±0.29ab	3.37±0.19ac	2.18±0.20b	2.82±0.29ab
**LDL-C (mmol/L)**	0.26±0.042a	0.45±0.09b	0.40±0.05b	0.22±0.06ab	0.384±0.13ab	0.36±0.11ab
**TC (mmol/L)**	2.43±0.15a	3.33±0.16b	2.83±0.35ab	3.28±0.11b	2.99±0.19b	3.23±0.11b
**TG (mmol/L)**	0.69±0.06a	1.13±0.00b	1.06±0.11b	1.17±0.08b	1.18±0.09b	1.45±0.21b
**UN (mmol/L)**	12.16±0.14a	13.23±0.31b	11.15±0.45ac	12.88±0.43abc	10.42±0.63ac	15.23±0.39c

After 4 weeks’ treatment, lipid levels were performed. The data were analyzed with ANOVA. The values are expressed as the mean ± SEM, n = 10. Columns labeled with different letters are significantly different at *P* < 0.05.

## 4. Discussion

According to data of Figs 1 and 2, high sugar high fat diet together with peritoneal injection of 60m/kg STZ induced experimental T2DM mice with stable body weight and blood glucose. After treatment with glibenclamide (a conventional medicine for T2DM), blood glucoses of T2DM mice gradually decreased. These data confirm that long-term high sugar/fat diet with STZ injection is a reasonable method for establishing an experimental T2DM mouse [52].

From Figs 2 and 3, we confirmed that extracts from *L*. *polystachyus* Rehd. leaves had hypoglycaemic effects. These effects were consistent with those of previous studies that showed flavanoids with MeOH-Butanol groups can improve the serum glucose level and glucose tolerance in STZ-induced hyperglycemic mice [19, 53]. Our results (Tables 1 and 2) were similar to Hou and Zhou, which showed that the antidiabetic ability of flavonoids was due to multiple mechanisms involved in blood lipid and antioxidant mediation [19], including attenuated leptin resistance in epididymal adipose tissue [54]. After four weeks of leaf extract treatment, our data show that flavonoids decrease blood glucose and maintain stable blood levels. Meanwhile, leaf extract treatment had a high utilization in body, which was similar to glibenclamide treatment. It is known that Chinese traditional medicine is a complex formulation which is typically metabolized in the liver. Zeng *et al*. (2010) showed that treatment with lower levels of total flavonoids (< 1g/kg·d) for short time periods (< 13 weeks) does not cause hepatic dysfunction [55]. Based on this work as well as [54], together with our preliminary research, the proper dose of leaf extract is 0.8g/kg/d.

We found increased hepatic glycogen contributed to the hypoglycemic effect in experimental T2DM mice (Fig 4). Martin Krssak *et al*. [56] showed that hepatic glycogen concentrations in T2DM patients were lower than control—these results were the same as the trends observed in our data. From Figs 6 through 8, we show that leaf extract treatment promotes hepatic glycogen through increasing glucose uptake and overexpression of genes relative to intracellular GK. Insulin has been shown to affect hepatic glucose flux via both direct and indirect mechanisms [57, 58]. Our results show that normal level insulin (5.0μg/mL insulin) directly affected glucose influx. In the liver, GK enhanced glycolysis, and was involved in hepatic glucose uptake which resulted in reduced blood glucose levels similar to other work [59]. Leaf extract treatment promoted overexpression of GLUT2 on the cell membrane that facilitated hepatic glucose influx and in turn increased hepatic glycogen concentration. Liu *et al*. [1] found that in addition to promoting intracellular GLUT4 translocation to the plasma membrane, resulting in enhanced glucose uptake, activation of Akt (protein kinase B) can phosphorylate GSK3β (glycogen synthasekinase-3), and lead to increased glucose transport [58].

Liver is a major insulin sensitive tissue, and responds to insulin-stimulated glucose uptake through PI3K/ PDK1/PKB signaling [60] and AMPK [61, 62] insulin signal transduction. IR initiates PI3K/ PDK1/PKB signaling, and depletion of hepatic IR significantly impairs downstream insulin signaling [57]. The activated IR led to tyrosine phosphorylation of IRS [61], and facilitated PI3K/ PDK1/PKB signaling, leading to phosphorylation of Ser/Thr activated kinase Akt (PKB). Combining these principles with Fig 8, we speculate that leaf extract treatment caused over-expression of IR and IRS, which increased insulin-sensitivity and insulin resistance by activating PI3K/ PDK1/PKB signaling. Over-expression of IR and IRS were evidence for hepatic glucose influx with 5.0 μg/mL insulin, leading to decreased blood glucose in T2DM mice.

Glucose homeostasis maintains not hepatic glycogen and also glycogenolysis. Ping *et al*. found transcription of PEPCK and G-6-Pase promoters were suppressed by activated MEK/ERK (Mitogen activated protein kinase-1/Extracellular signal-regulated kinase) [9]. Others have shown that palmitate treatment can increase PEPCK and G-6-Pase expression [5], while olanzapine treatment increases only G-6-Pase expression [63]. These previous works show that activation or overexpression of PEPCK and/or G-6-Pase contributes to hepatic glucose production, causing high blood glucose levels. Fig 8 shows that the mechanism by which leaf extract causes hypoglycemic effect is partially due to inhibition of PEPCK and/or G-6-Pase mediated hepatic glucose production. Consequently, leaf extract treatment may have consequences on liver functions, including over-activation of glycogenesis by increased glucose influx, overexpression of GK, GLUT2, IR and IRS and/or a decline in gluconeogenesis under fasting conditions by suppressing the expression of PEPCK and/or G-6-Pase.

Over 30 years ago, Wu *et al*. [64] isolated, purified, and identified dihydrochalcone from *L*. *polystachyus* Rehd. leaves, from tea for the first time. Later, trilobatin [65] and phloridzin [24] were identified in leaf extracts. The presence of SOD and GSH were confirmed over ten years ago [66], furthermore this work showed that trilobation has a better ability in reducing liver oxidative stress than phloridzin *in vitro*. As shown in Table 1, we found similar results for SOD, but different results for GSH *in vivo*. Recent work by Dong (2012) showed that trilobatin can inhibit α-glucosidase with less side effect than acarbose to manage postprandial hyperglycemia [21] and attenuate inflammatory response [67] *in vitro*. Until now, treatment with trilobatin from *L*. *polystachyus* Rehd. leaves *in vivo* has not been reported. Fig 1 shows that treatment with trilobatin has no effect on body weight, but Figs 4 and 6 shows a significant hypoglycemic effect, and no difference in hepatic glycogen when compared to phloridzin. Our data of glucose flux and mRNA expression shown increased expression of GK, GLUT2 and IRS with trilobatin treatment, but no marked increased in glucose influx. We hypothesized that activated IRS or a lack of activated IR deters activation of Akt, which then causes GLUT4 in the cell membrane to be exposed. Nevertheless, PEPCK was significantly overexpressed after trilobatin or phloridzin treatment, while only G-6-Pase was overexpressed only with phloridzin treatment. This may be the reason why trilobatin had antidiabetic effects *in vivo*. Furthermore, as shown in Table 2, we conclude that treatment with trilobatin causes over-expression of GK, expression of GLUT2 and IRS, also leads to a decline in liver oxidative stress and increase in lipid level.

Dong *et al*. found that phloridzin and phloridzin-6"-O-salicylate from *L*. *polystachyus* Rehd. leaves increased blood lipid levels and enhanced antioxidant activity in T2DM mice [68, 69], which was attributed to lower blood glucose levels. Flavonoids eliminate superoxide anion (O_2_^- ·^) by redox chemistry with the phenolic hydroxyl group. In spite of 2'-hydroxyl glycosylation [70], phloridzin has a higher comprehensive capacity in reducing liver oxidative stress than trilobatin. Dong *et al*. [68] found that sweet tea phloridzin significantly decreased MDA and increased SOD and GSH-Px in T2DM mice. Our results in Tables 1 and 2, verify this, showing that treatment with phloridzin leads to higher levels of T-SOD, GPT and MDA than T2DM, and improves lipid levels in experimental T2DM mice. Phloridzin a known inhibitor of SGLT-1 [22, 71] and SGLT-2 [72], and therefore glucose flux in the small intestine. In liver tissue, phloridzin also inhibited glucose flux (Figs 6 and 7), through GLUT2 inhibition, which confirms work by [71]. Although treatment with phloridzin increased expression of GLUT2, expression of similar genes (GK, IR and IRS) was reduced relative to leaf extract treatment, while overexpression of PEPCK and G-6-Pase was pronounced relative to leaf extract treatment. In summary, phloridzin inhibits glucose uptake in liver tissue, and the antidiabetic effect is likely due to higher capacity in reducing oxidative stress.

In this study, we found that leaf extract, trilobatin and phloridzin had a remarkable hypoglycemic effect caused by enhanced glucose uptake, hepatic glycogen synthesis, and liver glycogen while reducing hepatic gluconeogenesis and liver oxidative stress in long-term double high-fed and STZ-induced T2DM mice. For the first time, we have connected glycogen metabolism with glucose flux in liver tissue, correlating body weight, liver oxidative stress and lipid levels, and mRNA expression. Although we did not test the combinatorial effect of trilobatin and phloridzin, we show that leaf extract treatment has enhanced antidiabetic effects when compared to trilobatin and phloridzin alone.

## Supporting Information

S1 Fig The extraction process of *Lithocarpus polystachyus* Rehd., phloridzin and trilobatin. (TIF)

S2 Fig High resolution ^1^H- NMR 600 MHz spectra of phloridzin (A) and trilobatin (B). (TIF)

S3 Fig Leaf extracts from *L*. *polystachyus* Rehd. decreased blood glucose of experimental T2DM mice after gavage of different groups. Blood glucose of groups control (A), T2DM (B), glibenclamide (C), leaf extract (D), phloridzin (E) and trilobatin (F) were relatively unchanged. The data were analyzed using an ANOVA with repeated measures with a Sphericity Assumed or Greenhouse-Geisser correction, and the mean scores for body weight were statistically reported that control (*F* (1.255, 5.002) = 0.843, *P* = 0.429), T2DM (*F* (1.745, 17.538) = 1.821, *P* = 0.144), glibenclamide (*F* (1.723, 17.227) = 4.331, *P* = 0.035), leaf extract (*F* (1.779, 17.79) = 3.437, *P* = 0.059), phloridzin (*F* (1.511, 13.595) = 1.002, *P* = 0.37) and trilobatin (*F* (4, 40) = 1.638, *P* = 0.184), respectively. All values are expressed as the mean ± SEM, n = 10. Columns labeled with different letters are significantly different at *P* < 0.05.
(TIF)

S4 Fig The net basal influx of glucose and the fitted curve of control group mice. For this control mouse sample, the oscillation period was 4.54 min.
(TIF)

S1 Table Formula of high sugar & high fat mice feed. (DOCX)

S2 Table Primer Sequences of Genes. (DOCX)
