# Supplementary figures and images for: Leaf Extract from Lithocarpus polystachyus Rehd. Promote Glycogen Synthesis in T2DM Mice
Source: PLoS One. 2016 Nov 28;11(11):e0166557. doi: 10.1371/journal.pone.0166557 (PMC5125604; doi:10.1371/journal.pone.0166557)

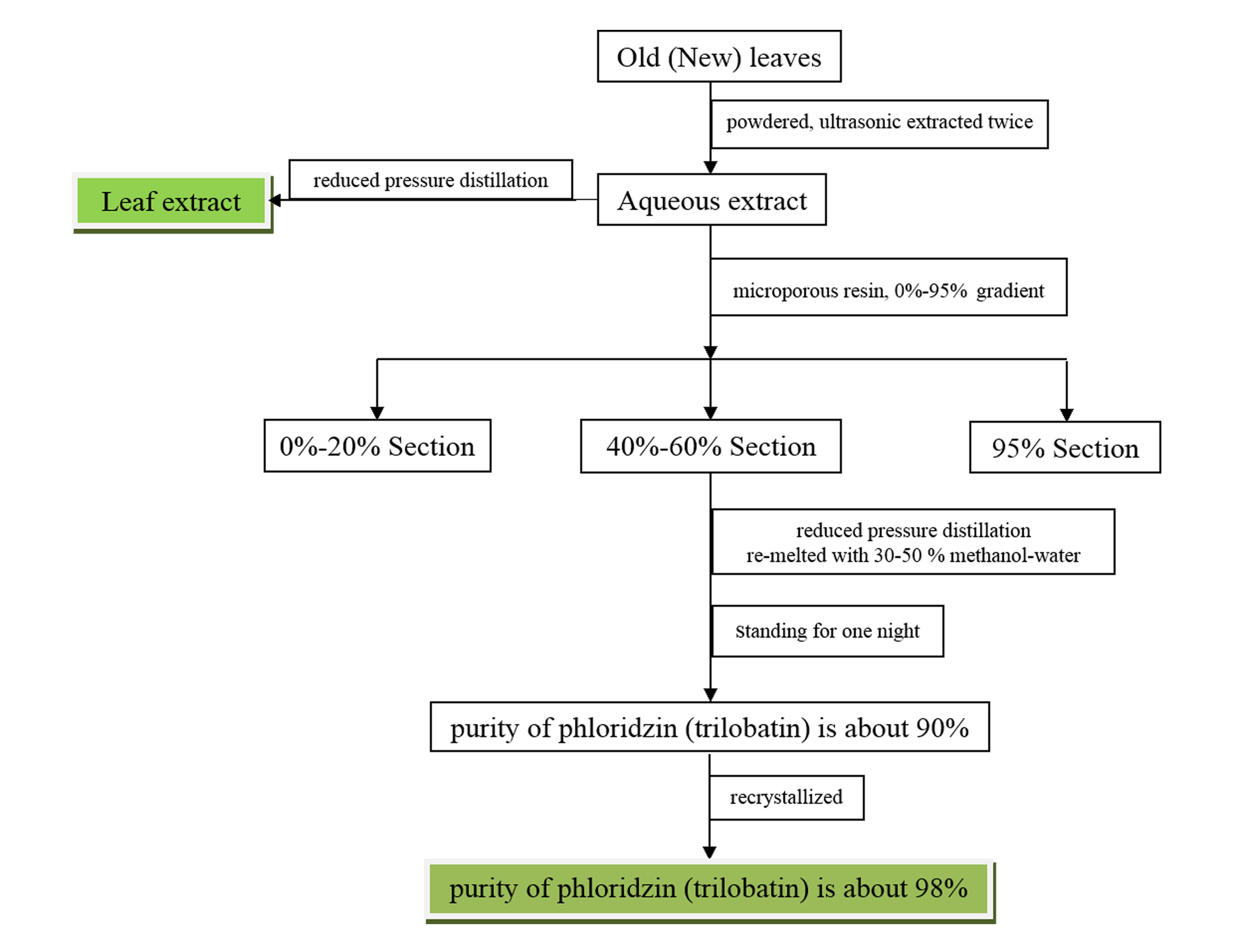

Supplement: S1 Fig — (TIF) [file pone.0166557.s001.tif]

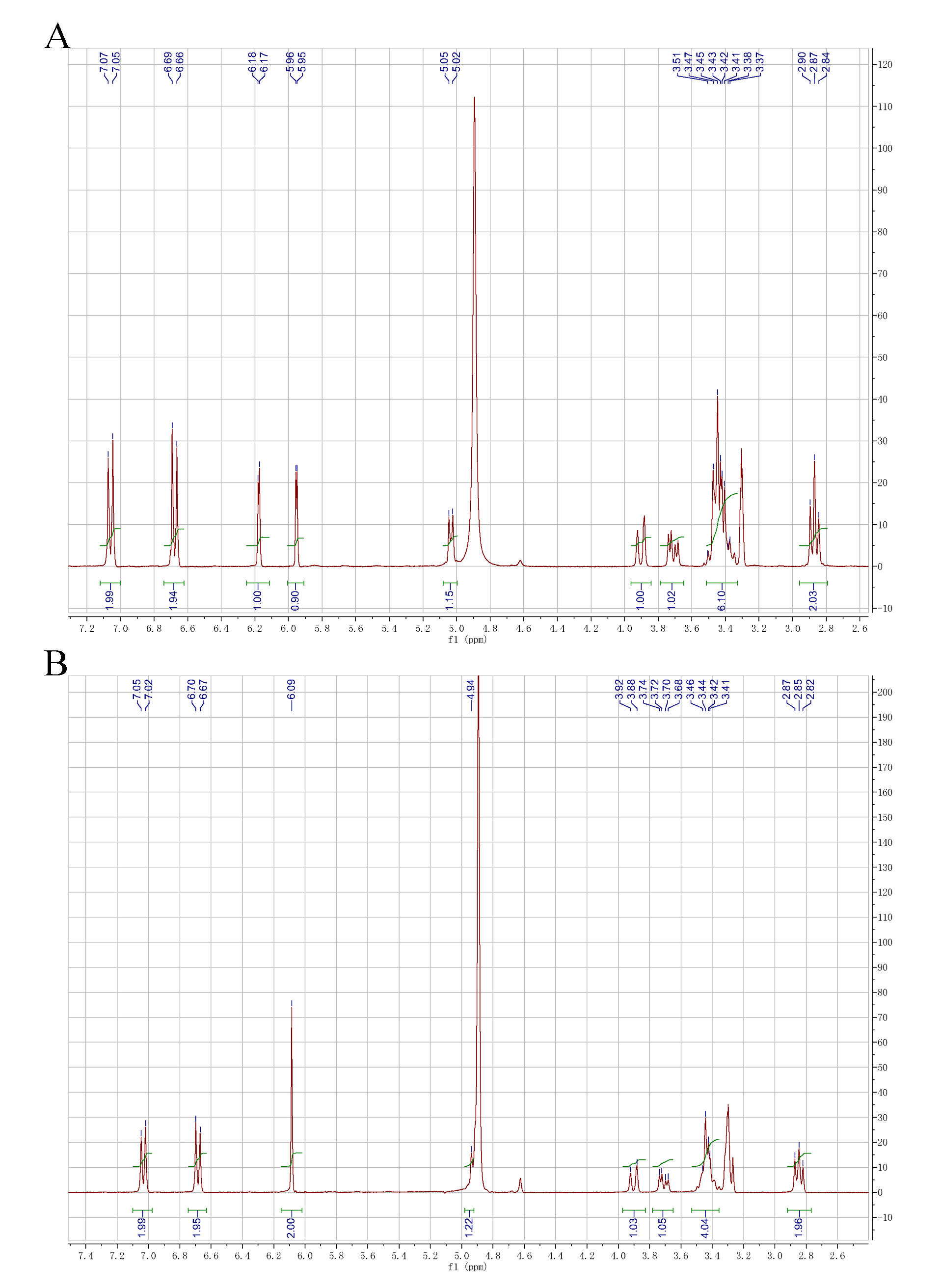

Supplement: S2 Fig — (TIF) [file pone.0166557.s002.tif]

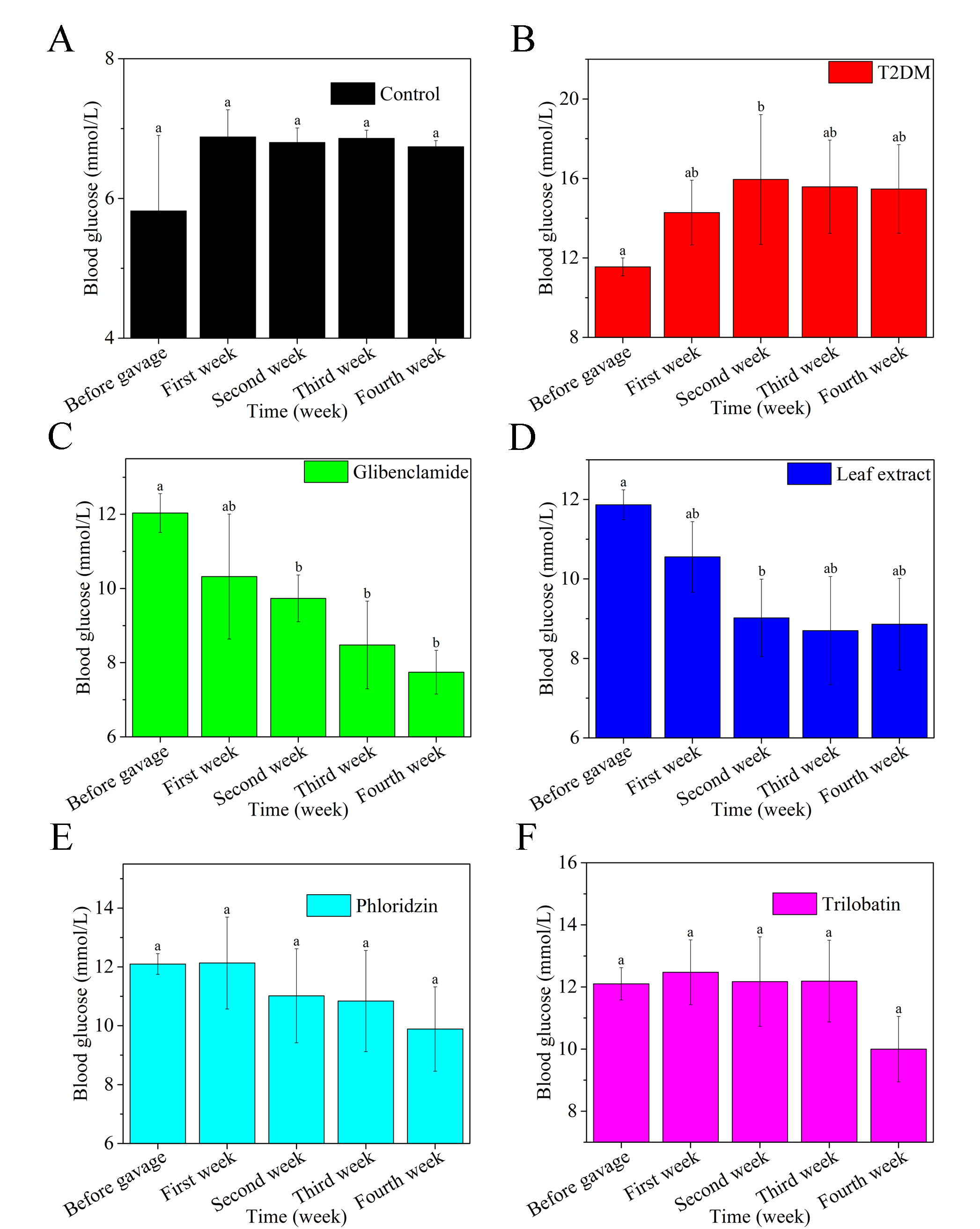

Supplement: S3 Fig — Blood glucose of groups control (A), T2DM (B), glibenclamide (C), leaf extract (D), phloridzin (E) and trilobatin (F) were relatively unchanged. The data were analyzed using an ANOVA with repeated measures with a Sphericity Assumed or Greenhouse-Geisser correction, and the mean scores for body weight were statistically reported that control (F (1.255, 5.002) = 0.843, P = 0.429), T2DM (F (1.745, 17.538) = 1.821, P = 0.144), glibenclamide (F (1.723, 17.227) = 4.331, P = 0.035), leaf extract (F (1.779, 17.79) = 3.437, P = 0.059), phloridzin (F (1.511, 13.595) = 1.002, P = 0.37) and trilobatin (F (4, 40) = 1.638, P = 0.184), respectively. All values are expressed as the mean ± SEM, n = 10. Columns labeled with different letters are significantly different at P < 0.05. (TIF) [file pone.0166557.s003.tif]

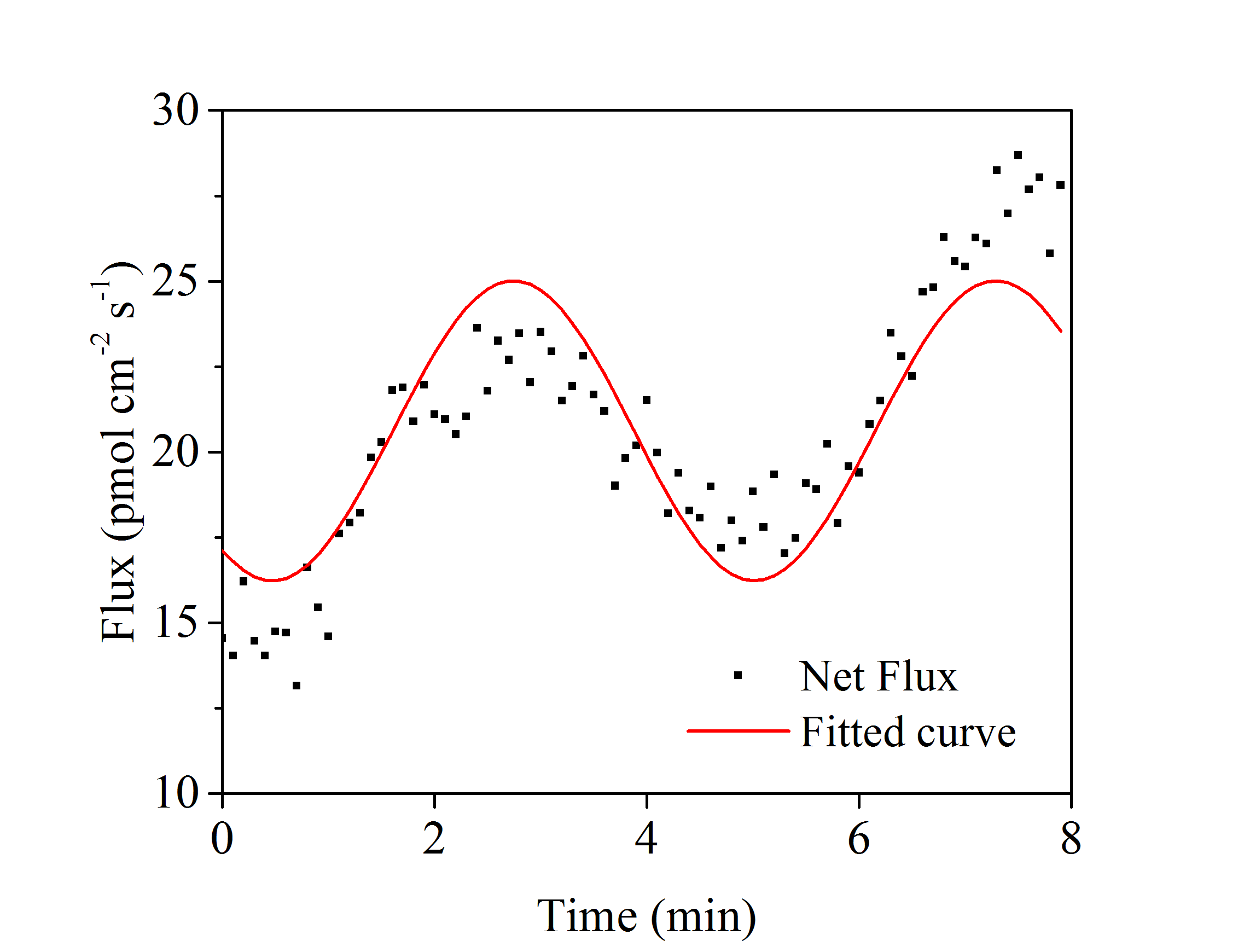

Supplement: S4 Fig — For this control mouse sample, the oscillation period was 4.54 min. (TIF) [file pone.0166557.s004.tif]
