# Supplementary material for: Leaf Extract from Lithocarpus polystachyus Rehd. Promote Glycogen Synthesis in T2DM Mice
Source: PLoS One. 2016 Nov 28;11(11):e0166557. doi: 10.1371/journal.pone.0166557 (PMC5125604; doi:10.1371/journal.pone.0166557)
Supplement: S1 Table — (DOCX) [file pone.0166557.s005.docx]

**S1 Table: Formula of high sugar & high fat mice feed**

| Name | Share (%) |
| --- | --- |
| Conventional feed | 66.5 |
| Lard | 10.0 |
| Saccharose | 20.0 |
| Cholesterol | 2.5 |
| Cholate | 1.0 |
