# Supplementary material for: Leaf Extract from Lithocarpus polystachyus Rehd. Promote Glycogen Synthesis in T2DM Mice
Source: PLoS One. 2016 Nov 28;11(11):e0166557. doi: 10.1371/journal.pone.0166557 (PMC5125604; doi:10.1371/journal.pone.0166557)
Supplement: S2 Table — (DOCX) [file pone.0166557.s006.docx]

**S2 Table: Primer Sequences of Genes**

| Name | Sequences (5'-3') |
| --- | --- |
| *GK-F* | TAAAGATGTTGCCCACCTACG |
| *GK-R* | GGAATACATCTGGTGTTTCGTCT |
| *G-6-p-F* | ATCAATCTCCTCTGGGTGGC |
| *G-6-p-R* | TGTTGCTGTAGTAGTCGGTGTCC |
| *GLUT-2-F* | TGGCTCGGGGACAAACTT |
| *GLUT-2-R* | AGCAATGATGAGGGCGTGT |
| *IR-F* | TGGGCTTCGGGAGAGGA |
| *IR-R* | GGATGTCCATACCAGGGCAC |
| *IRS1-F* | CAAGGAGGTCTGGCAGGTTA |
| *IRS1-R* | CCCACCTCGATGAAGAAGAA |
| *PEPCK-F* | GAGAAAGCATTCAACGCCAGG |
| *PEPCK-R* | CACAGATATGCCCATCCGAGTC |
| *GADPH-F* | ACCACAGTCCATGCCATCAC |
| *GADPH-R* | TCCACCACCCTGTTGCTGTA |
